# A Comparative Analysis of U-Net and Vision Transformer Architectures in Semi-Supervised Prostate Zonal Segmentation

**DOI:** 10.3390/bioengineering11090865

**Published:** 2024-08-26

**Authors:** Guantian Huang, Bixuan Xia, Haoming Zhuang, Bohan Yan, Cheng Wei, Shouliang Qi, Wei Qian, Dianning He

**Affiliations:** 1 College of Medicine and Biological Information Engineering, Northeastern University, Shenyang 110057, China; 2271324@stu.neu.edu.cn (G.H.); 2271354@stu.neu.edu.cn (B.X.); 20207364@stu.neu.edu.cn (B.Y.); wqian@bmie.neu.edu.cn (W.Q.); 2 School of Science and Engineering, University of Dundee, Dundee DD1 4HN, UK; c.wei@dundee.ac.uk; 3 School of Health Management, China Medical University, No. 77 Puhe Road Shenyang North New Area, Shenyang 110122, China

**Keywords:** comparative analysis, prostate zonal segmentation, semi-supervised learning, U-Net, vision transformer

## Abstract

The precise segmentation of different regions of the prostate is crucial in the diagnosis and treatment of prostate-related diseases. However, the scarcity of labeled prostate data poses a challenge for the accurate segmentation of its different regions. We perform the segmentation of different regions of the prostate using U-Net- and Vision Transformer (ViT)-based architectures. We use five semi-supervised learning methods, including entropy minimization, cross pseudo-supervision, mean teacher, uncertainty-aware mean teacher (UAMT), and interpolation consistency training (ICT) to compare the results with the state-of-the-art prostate semi-supervised segmentation network uncertainty-aware temporal self-learning (UATS). The UAMT method improves the prostate segmentation accuracy and provides stable prostate region segmentation results. ICT plays a more stable role in the prostate region segmentation results, which provides strong support for the medical image segmentation task, and demonstrates the robustness of U-Net for medical image segmentation. UATS is still more applicable to the U-Net backbone and has a very significant effect on a positive prediction rate. However, the performance of ViT in combination with semi-supervision still requires further optimization. This comparative analysis applies various semi-supervised learning methods to prostate zonal segmentation. It guides future prostate segmentation developments and offers insights into utilizing limited labeled data in medical imaging.

## 1. Introduction

With the massive increase in annotated data, deep learning has achieved significant success in image segmentation. However, the acquisition of annotated medical image data is often expensive because generating accurate annotations requires expertise and time. The accuracy of medical image segmentation in specific areas such as the prostate is crucial [1]. In recent years, multiparametric magnetic resonance imaging (mpMRI) has provided an important analytical basis for the detection and staging of prostate cancer [2]. Most of the current analyses are based on tumor detection and analysis but ignore the importance of structural information in different anatomical regions of the prostate for quantitative analysis of the prostate [3]. The prostate consists of four anatomical zones: the transition zone (TZ), peripheral zone (PZ), central zone (CZ), and anterior fibromuscular stroma (AFS) [4]. The clinical importance of these regions is mainly in the diagnosis and treatment of prostate diseases. For example, prostate cancer often occurs in the PZ, whereas benign prostatic hyperplasia occurs mainly in the TZ [5], and the location of the AFS also constitutes an important guide for prostate surgery [6]. Owing to the complex morphology of the four prostate regions, there are currently few annotated data and an increasing amount of unlabeled prostate image data, which poses challenges to the accurate segmentation of prostate regions.

Prior to the rise of deep learning, prostate region segmentation was addressed by methods such as an approach to atlas matching using local mutual information [7] and feature pattern recognition [8]. Classical methods do present significant challenges in distinguishing internal regions of the prostate. Conventional edge detection algorithms usually rely on binarization, an approach that has limitations in multi-region segmentation tasks. Following the success of U-Net [9], supervised learning has been proposed to employ variants of U-Net to segment the prostate as a whole structure [10], or simply segment it into PZ and TZ [11]. Robin et al. [12] proposed a novel approach for semantic segmentation based on Vision Transformer, which did not use convolution and was successful in semantic segmentation by capturing contextual information by design.

After the success of deep learning in the field of segmentation, current prostate-based segmentation methods only detect the entire prostate region or simply divide it into PZ and TZ [13]. However, these methods do not take into account internal structures that are difficult to distinguish, such as the small amount of AFS and distal prostatic urethra (DPU). When a more detailed segmentation of the prostate is needed, it becomes more challenging due to the limited number of labeled data for deep learning model development.

Karimi et al. [14] learned subtle patterns of prostate shape changes in a process known as statistical shape modeling in order to overcome the lack of training data for prostate images. This strategy did not guarantee that all the generated data correspond exactly to the labels. Semi-supervised learning using unlabeled data is therefore more in line with the current state of medical images than generated data. So, we selected five semi-supervised learning models, namely, entropy minimization (EM) [15], mean teacher (MT) [16], uncertainty-aware mean teacher (UAMT) [17], cross pseudo-supervision (CPS) [18], and interpolation consistency training (ICT) [19]. We used the Dice [20] and true positive rate (TPR) [21] indicators to evaluate these experiments and compared the outcomes with the results of the current state-of-the-art (SOTA) prostate semi-supervised network uncertainty-aware temporal self-learning (UATS) [22].

Semi-supervised learning has an advantage in that it can not only make use of the powerful feature extraction capabilities of deep learning models, but can also use unlabeled data to improve model generalization [23]. EM was initially proposed as an extremely simple and effective method [24], which demonstrated that minimizing the entropy of the prediction over unlabeled data could improve model performance and inspired many subsequent works [25]. Pseudo-label trains an initial model on labeled data and uses the unlabeled data to infer in order to generate pseudo-labels and iteratively further trains using the pseudo-label [26]. CPS took advantage of this feature and achieved good performances in semantic segmentation. This idea was also extended to some new semi-supervised learning models [27].

Consistency regularization training is the most commonly used semi-supervised learning method in the field of deep learning, which perturbs or augments the inputs and applies consistency so that the model produces similar outputs for them. MT was an algorithm proposed for temporal ensemble [28] with a large computational cost to improve on the weights of the model. MT achieved good performances in natural image classification. Continuing this idea of MT, there has been much subsequent work in medical imagery [29]. UAMT achieved good performances in natural image classification by Monte Carlo Dropout to estimate the uncertainty of each target prediction to improve the performance of MT. UAMT was guided by estimation uncertainty, and unreliable predictions were filtered out when calculating the loss of consistency. UAMT was based on 3D left lung images for medical image segmentation. This task only required segmentation of the foreground and background and has not been applied to prostate zonal segmentation. To prevent the problem that adversarial perturbation training impairs the generalization performance, ICT used interpolation to improve MT in natural image classification.

Specifically, EM achieves semi-supervised learning by jointly training supervised segmentation loss and unsupervised entropy loss. CPS employs models with different initializations but the same network structure for natural image segmentation and achieves model consistency through pseudo-label consistency loss computation. The MT targets natural image classification and uses average model weights rather than label predictions to improve the training and testing accuracy through fewer labels. UAMT focuses on left lung segmentation and estimates the uncertainty of the target prediction through Monte Carlo Dropout to improve the training and testing accuracy by filtering unreliable predictions to improve the reliability of the student model. ICT overcomes the impairment of generalization performance by adversarial perturbation training, with particular applications in natural landscape classification. UATS combines the concepts of self-learning and temporal integration to improve the accuracy of segmentation of different regions of the prostate. We explore the impact of multiple semi-supervised models on prostate image segmentation using U-Net and the natural image segmentation method Vision Transformer (ViT) [12] as backbones.

MT and ICT were applied to natural image classification, CPS was applied to the semantic segmentation of natural images, and UAMT was applied to medical images with only foreground and background. These methods have not been tried on segmenting regions of prostate images. Therefore, we put these five representative semi-supervised learning methods into ViT as well as U-Net backbones and compared them with the temporal ensemble-based SOTA method UATS for semi-supervised segmentation methods for the prostate region. This allows for a more comprehensive analysis of the capabilities of semi-supervised learning in the field of prostate segmentation.

This comparative analysis presents valuable insights into the relative strengths and limitations of the U-Net and ViT architectures for semi-supervised prostate zonal segmentation. The subdivided regions of the prostate are challenging and therefore their segmentation is not an easy task to accomplish. By investigating the details of their performance on the prostate dataset, this comparative analysis is expected to provide new understanding and methods for semi-supervised learning in other areas of medical image segmentation with complex structures.

## 2. Materials and Methods

### 2.1. Dataset and Pre-Processing

For the prostate region segmentation, we used the ProstateX dataset [30], which provided publicly available ground truth annotations introduced by Meyer et al. [31]. The dataset contained multisite prostate MRI scans of healthy individuals, patients with cancer, and patients with hyperplasia under a variety of conditions. The dataset contained 346 T2w axial volumes. Of these, 98 volumes were associated with labels for the PZ, TZ, AFS, and DPU. Importantly, 248 masses were unlabeled to facilitate our semi-supervised learning strategy. We randomly selected 58 labeled samples as the training set, with the validation and test sets each containing 20 samples. During the semi-supervised training, we added 248 unlabeled patient samples to the training set. To investigate whether additional labeled data would benefit the semi-supervised model, we randomly split the 20 test samples into two groups. One group continued to serve as the test set, while the other group was added to the training set. The groups were then swapped, and the experiment was repeated.

The raw volumes showed different resolutions in the range of [0.3–0.6] × [0.3–0.6] × [3.0–5.0] mm. To establish consistency in the input dimensions, we resampled all the volumes to a common spacing of 0.5 × 0.5 × 3 mm to adjust the spatial resolution of an image so that it had the same pixel spacing to achieve a more accurate segmentation. For the labels, we used the closest interpolation to ensure label integrity. These 3D volumes were cropped into 2D images with a fixed size of 224 × 224 pixels to normalize the input dimensions and reduce possible noise or irrelevant information. The intensity values were normalized to fall within the [0, 1] interval. This reduced sensitivity to changes in the distribution of the input data, which helped the model learn features more efficiently and updated weights more stably during the training process.

### 2.2. Supervised Learning Methods

#### 2.2.1. U-Net

**Figure 1:**
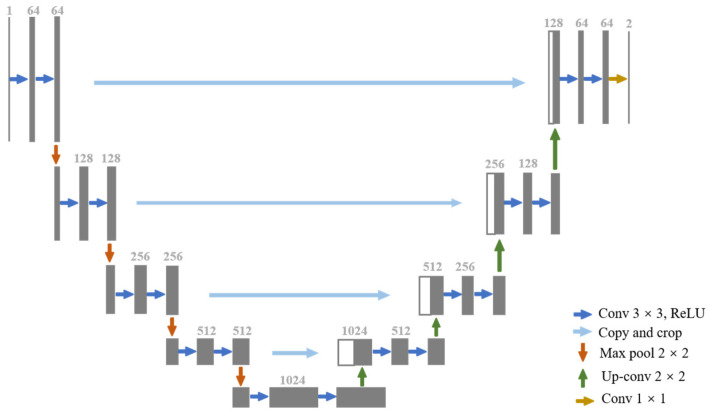
Overview of U-Net framework. The grey box indicates a multi-channel feature map, with the number of channels marked at the top of the box and the dimensions in the bottom left corner. The white box indicates a copied feature map, and the arrows indicate different operations.

As shown in Figure 1, U-Net, which is renowned for its outstanding performance in medical image segmentation, features an encoder–decoder structure. We fine-tune the U-Net parameters, including the learning rates and weight decay, to adapt them to our segmentation task. The encoder–decoder structure facilitates the extraction of both local and global features in the images.

#### 2.2.2. Vision Transformer

**Figure 2:**
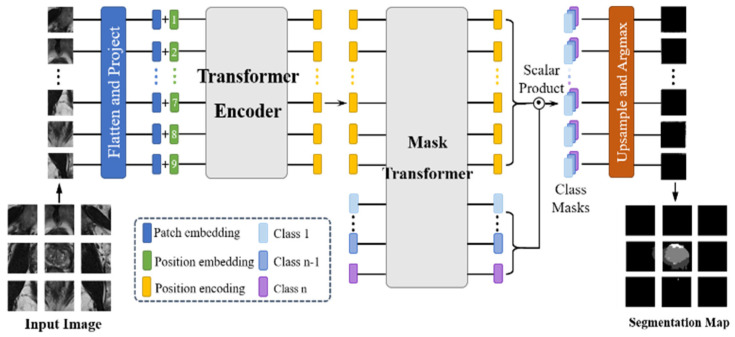
Overview of Vision Transformer framework. Transformer Encoder: The image patches are projected onto a sequence of embeddings and then encoded with a transformer. Decoder: A mask transformer takes as input the output of the encoder and class embeddings to predict segmentation masks [12].

As shown in Figure 2, the ViT approach first divides the input image into patch embeddings and uses position embeddings to correspond to the patch embeddings. The position embeddings are fed into the transformer encoder, which uses its self-attention mechanism to capture the relationships between image blocks and generates sequential encoding filled with global contextual information. The decoding stage uses a mask transformer specifically tailored to image segmentation, which learns pixel relationships and semantic information to produce a segmented output corresponding to the input image. The final step consists of the decoder output and applying class embeddings to assign class labels to each pixel to generate a final pixel-by-pixel segmentation map.

### 2.3. Semi-Supervised Learning Methods

#### 2.3.1. Entropy Minimization

EM increases confidence in the predictions of unlabeled data by reducing the entropy of the model output and increasing confidence in the overall model output using unlabeled data.

The regularization term, denoted as $L_{entropy}(p)$, is mathematically expressed as:

(1) $$L_{entropy}(p)=-\frac{1}{log(C)}\sum_{i=1}^{C}p_{i}log(p_{i}+\epsilon )$$

Here, *C* represents the number of classes, $p_{i}$ signifies the predicted probability for class *i*, and *ϵ* is a small constant introduced to prevent numerical instability.

#### 2.3.2. Cross Pseudo-Supervision

**Figure 3:**
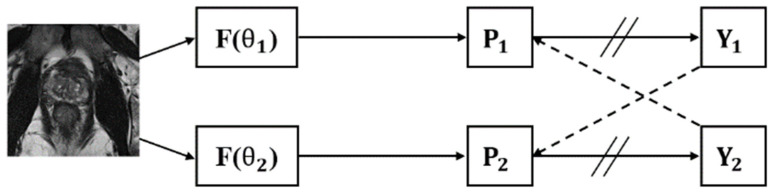
Overview of cross pseudo-supervision framework. The F stands for backbone and the double slash on the arrow stands for integration.

In the CPS framework shown in Figure 3, P1 and P2 are derived from the same input image. P1 and P2 generate labels Y1 and Y2, respectively, using argmax. Among these, Y2 acts as the supervisor of P1 and Y1 supervises P2.

The CPS framework predicts unlabeled data through models with the same network structure but different initialization parameters and then uses the prediction results of different models to calculate the losses for each other. CPS exploits the differences between model parameters to improve the adaptability of semi-supervised segmentation tasks.

#### 2.3.3. Mean Teacher

**Figure 4:**
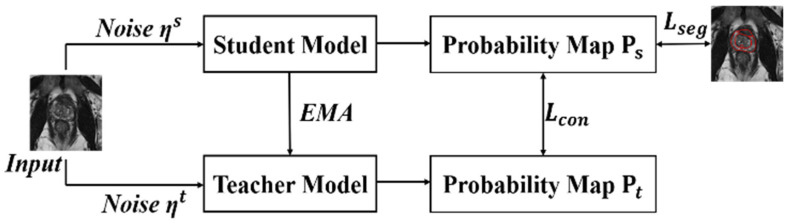
Overview of mean teacher framework. The student model represents the original backbone.

As shown in Figure 4, the MT framework divides the model into teachers and students. The teacher model is used to generate learning goals for students, and the student model uses the goals provided by the teacher for learning. The weight of the teacher model is obtained from the weighted average of the student model’s time memory. It is believed that when a small amount of perturbation noise is added to the input data, the prediction results of the model do not change.

In the context of the MT, the exponential moving average (EMA) mechanism is employed for parameter updates. The updated formula is expressed as

(2) $$\theta_{t}^{\prime }=\alpha \theta_{t-1}^{\prime }+(1-\alpha )\theta_{t}$$

where *α* signifies momentum, $\theta_{t}^{\prime }$ is the teacher network, and *θ* is the student network. For instance, when *α* is set to 0.95, the teacher network retains 95% of its parameters unchanged during each update, incorporating 5% from the student network.

#### 2.3.4. Uncertainty-Aware Mean Teacher

**Figure 5:**
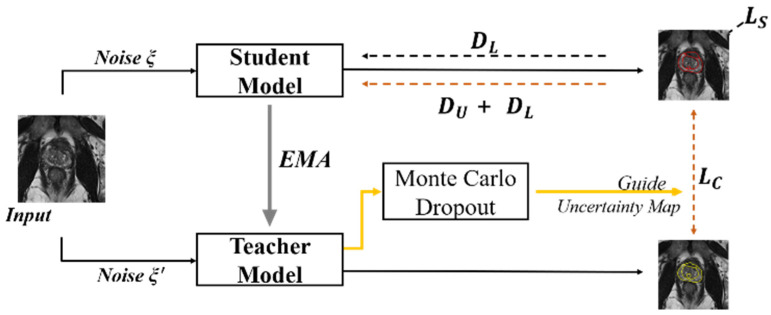
Overview of uncertainty-aware mean teacher framework.

In the UAMT framework shown in Figure 5, both the teacher and student models share identical network structures. The teacher model parameters are updated through the EMA of the student model parameters. The student model is optimized by minimizing both the supervised loss (*L_s_*) of labeled data (*D_L_*) and consistency loss (*L_C_*) of both the unlabeled data (*D_U_*) and labeled data.

During training, the teacher model not only generates target outputs but also estimates the uncertainty of each target prediction through Monte Carlo Dropout. Under the guidance of Monte Carlo Dropout, unreliable predictions are filtered out, and only reliable predictions are retained when calculating the consistency loss.

#### 2.3.5. Interpolation Consistency Training

**Figure 6:**
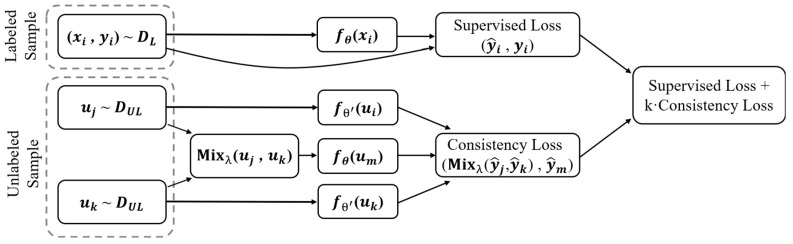
Overview of interpolation consistency training framework. *θ* represents the student model; *θ*′ represents the student model.

The ICT framework shown in Figure 6 involves interpolation between two distinct transformations of the input data, compelling the model to maintain consistency in its predictions. ICT leverages this concept by generating additional training samples through the interpolation and utilization of both labeled and unlabeled data. The consistency constraints imposed on these interpolated samples enable the model to adapt better to the distribution of unlabeled data, ultimately enhancing its performance in semi-supervised scenarios.

The parameter θ receives updates at each iteration t through stochastic gradient descent to minimize the loss function L, defined as the sum of the cross-entropy loss ($L_{S}$) on the labeled samples and a weighted consistency regularization loss ($L_{US}$). Both losses are computed on the mini-batch, and the weight w(t) is incrementally increased after each iteration. This incremental increases in w(t) amplifies the significance of the consistency regularization loss, aiding the model in effectively capturing and maintaining consistency in its predictions.

The formula for the interpolation operation on unlabeled data awakening is:

(3) $${Mix}_{\lambda }\left(a,b\right)=\lambda a+(1-\lambda )b$$

where two inputs, *a* and *b*, are linearly interpolated based on a mixing coefficient, λ.

ICT trains a prediction model, $f_{\theta }$, to provide consistent predictions at the interpolations of unlabeled points:

(4) $$f_{\theta }(Mix(u_{j},u_{k}))\approx {Mix}_{\lambda }(f_{\theta^{\prime }}\left(u_{j}\right),f_{\theta^{\prime }}\left(u_{k}\right))$$

where *θ*′ is a moving average of *θ*, $f_{\theta }(Mix(u_{j},u_{k}))$ is the prediction of the model $f_{\theta }$ on the mixup of unlabeled points $u_{j}$ and $u_{k}$, and ${Mix}_{\lambda }(f_{\theta^{\prime }}\left(u_{j}\right),f_{\theta^{\prime }}\left(u_{k}\right))$ is the mixup of predictions generated by the MT model *f_θ′_* on the same unlabeled points.

#### 2.3.6. Uncertainty-Aware Temporal Self-Learning

**Figure 7:**
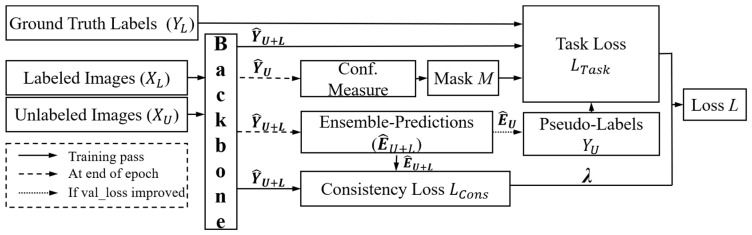
Overview of uncertainly-aware temporal self-learning framework.

As shown in Figure 7, UATS combines two semi-supervised learning techniques, self-learning and temporal integration. The idea of self-learning is to iteratively obtain improved models by extending the dataset. In order to limit the impact of erroneous pseudo-labels, the most plausible prediction is selected based on an uncertainty metric. Also, some concepts derived from temporal integration are incorporated, where the pseudo-label is updated based on the integrated prediction rather than the current period prediction. In addition, the loss of consistency between current and integrated forecasts is computed, enforcing consistency between current and prior period forecasts.

The consistency loss is obtained by computing the dissimilarity between the integrated prediction and the current network prediction. In the original spatio-temporal ensemble approach designed for classification, the similarity was measured by the mean square error. For the segmentation task, we find it more effective to define the similarity of the two segmentation results, as it is less sensitive to category imbalance and can cope with probabilistic segmentation.

### 2.4. Training Settings

We standardized the initialization parameters of U-Net and ViT to better compare the semi-supervised performances of the two models. In the semi-supervised learning, the batch sizes for both labeled and unlabeled data were experientially set to 12, and the initial learning rate was set to 0.005. The SGD was used to optimize model convergence. We combined the cross-entropy (CE) loss and Dice loss in the supervision part. We defined the total loss of the supervised part as 0.5 × (CE loss + Dice loss). The model learned features through the classification information of the CE loss and the segmentation accuracy of the Dice loss.

## 3. Results

### 3.1. Semi-Supervised Learning Methods

It should provide a concise and precise description of the experimental results and their interpretation, as well as the experimental conclusions that can be drawn.

Dice and TPR using supervised and semi-supervised segmentation methods are listed in Table 1. To comprehensively evaluate the overall performance of the model, we calculate the mean values of the overall Dice and TPR.

In supervised learning, the performance of U-Net is superior to that of ViT. In the TZ area, which has a large number of pixels and a relatively regular segmentation area, Dice reaches 85.99% and TPR reaches 85.01%. U-Net demonstrates consistent performance in areas with a large number of pixels, such as the PZ and TZ, as well as in areas with fewer pixels, such as the DPU and AFS, showing that U-Net can demonstrate the accuracy of its segmentation with a small amount of labeled data. On the other hand, ViT only parallels the performance of U-Net in the TZ area, where the number of pixels is larger, and the segmentation area is more regular. The segmentation performance of ViT in the rest of the prostate zones and the overall performance are affected by limited data and the large gap between medical and natural images.

To assess the improvement in U-Net and ViT after incorporating semi-supervised learning intuitively, we calculated the growth percentage of the four prostate regions and the overall mean values compared with supervised learning, as shown in Table 2.

**Figure 8:**
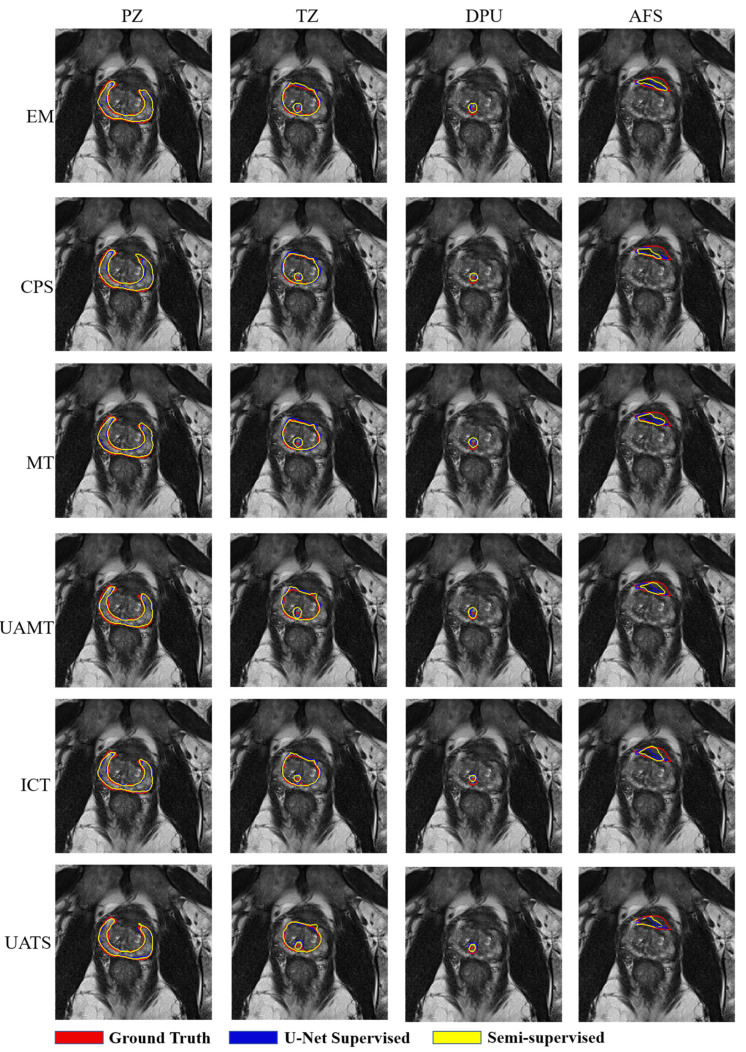
Four prostate zones are segmented based on U-Net with 58 patients used for training. The red contour represents the ground truth, the blue contour represents the result of segmentation using U-Net supervised learning, and the yellow contour is the result of segmentation using semi-supervised learning.

Figure 8 illustrates the comparison and supervised learning segmentation results with U-Net as the backbone. The supervised and semi-supervised segmentation effects of U-Net are relatively good, and the segmentation edges are smoother.

In the experiments using U-Net as the backbone, we observe the impact of different methods on the model performance. Specifically, EM shows a significant improvement in the DPU area, where Dice and TPR increase by 2.56% and 4.46%, respectively. In the AFS area, Dice increases by 6.38%, whereas TPR decreases by 0.73%. CPS achieves remarkable results in the PZ area, where Dice and TPR increase by 4.03% and 11.03%, respectively. In the AFS area, Dice and TPR increase by 8.94% and 7.93%. However, in the DPU area, Dice increases by 6.38%, whereas TPR decreases by 0.44%. The MT achieves good results in the DPU area, with Dice and TPR increasing by 2.95% and 6.08%. In the TZ, Dice increases by 6.38%, but TPR decreases by 0.11%. The UAMT shows a significant improvement in TPR, with an increase of 3.99% in the TZ area, an increase of 9.08% in the DPU area, and an overall performance improvement of 9.08%. The ICT achieves a 2.98% increase in Dice and a 6.01% increase in TPR in the PZ area. In the AFS area, Dice and TPR increase by 12.37% and 12.48%. Overall, the performance improvs by 3.55% for Dice and 4.61% for TPR. UATS provides a significant improvement in TPR in every area, with an average TPR improvement of up to 4.77% in the aggregate.

Using ViT as the backbone, the CPS exhibits a slight overall improvement, with Dice increasing 0.05% and TPR increasing 3.63%. For the UAMT, the TPR improvement effect is more significant, with an increase of 2.62% in the TZ area, 20.7% in the AFS area, and an overall increase of 4.53%. ICT improves in terms of overall performance, with increases in Dice and TPR in each region.

**Figure 9:**
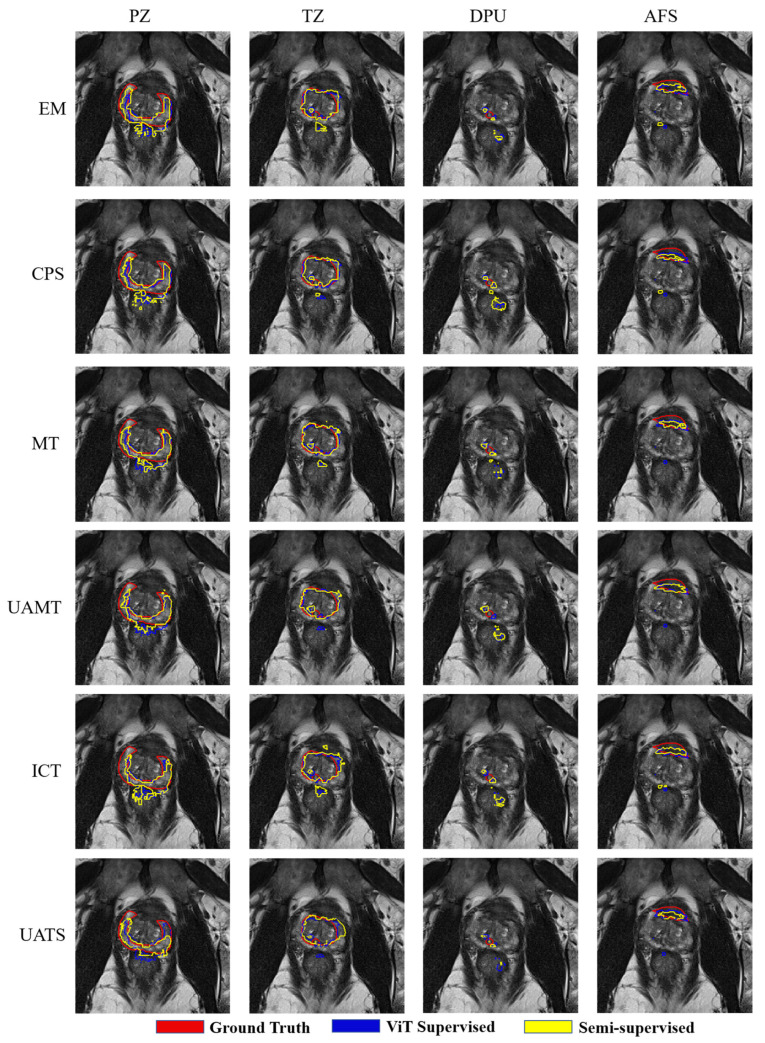
Four prostate zones are segmented based on ViT with 58 patients used for training. The red contour represents the ground truth, the blue contour represents the result of segmentation using ViT supervised learning, and the yellow contour is the result of segmentation using semi-supervised learning.

Figure 9 shows the segmentation results with ViT as the backbone. It can be seen that when the prostate image is segmented into different patches and put into the network under the ViT network, the tissue under the prostate is misinterpreted as prostate and the edges of the segmentation result are very rough.

To verify the effect of adding labeled data on the segmentation results, we randomly divided the 20 test patients into two groups and included them in the training and test sets, respectively. Subsequently, we switched the order and performed the experiment again. The test results are shown in Table 3. Comparing Table 2 and Table 3, we can find that when U-Net is used as the backbone, the addition of labeled data significantly improves the segmentation results, but semi-supervised learning decreases the increase in segmentation results compared to supervised learning. However, when using ViT as the backbone, adding labeled data is still less effective.

### 3.2. Classical Methods

To explore the advantages of deep learning comparison with classical segmentation methods, we used the traditional multi-category segmentation methods Markov Random Fields (MRF) [32], Mean Shift [33], and Ostu [34] for comparison. Table 4 shows the results of the classical method segmentation. Comparing it with Table 2, it can be seen that the segmentation accuracy of the classical method in the four regions is much lower than that of the deep learning method. In addition, the classical method is almost unrecognizable in the DPU area (targets with a small number of pixels and surrounded by other tissues). This further illustrates the superiority of deep learning methods in handling complex medical image segmentation tasks.

**Figure 10:**
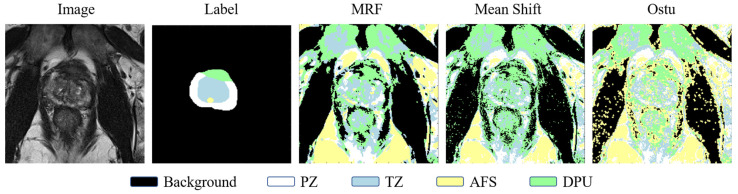
Four prostate zones are segmented based on classical methods.

Figure 10 shows the segmentation results of the three classical learning methods. It is obvious from the figure that the surrounding tissues have a very strong influence on the segmentation of the prostate. Specifically, the classical methods show significant limitations in dealing with the complex tissue structure around the prostate. The interference of the surrounding tissues led to inaccurate segmentation results, especially in the border region of the prostate, where the classical method was susceptible to the influence of the surrounding tissues, resulting in imprecise segmentation. These results further indicate that the performance of traditional segmentation methods is far inferior to modern deep learning methods when dealing with complex medical images.

## 4. Discussion

As medical datasets expand, a noteworthy amount of data from specialized fields such as prostate imaging still remains unlabeled. Classical edge extraction [35] methods are largely suitable only for binary segmentation tasks, while traditional multi-region segmentation approaches like MRF, Mean Shift, and Ostu are highly susceptible to interference from surrounding tissues when applied to prostate imaging. Consequently, using deep learning for prostate segmentation has become a prominent area of research. To address this problem, semi-supervised learning has attracted a lot of attention in the computer vision community, especially in the field of medical image analysis [23].

This comparative analysis presents a comprehensive evaluation of the U-Net and ViT architectures as the backbone of semi-supervised medical image segmentation on the ProstateX dataset. We highlight the significant performance differences in semi-supervised learning methods and reveal the unique advantages and limitations of each architecture. U-Net consistently outperforms ViT across all regions for the baseline tasks, as evidenced by the higher Dice coefficients. This demonstrates the remarkable flexibility and competence of U-Net when applied to medical image segmentation tasks [36].

In U-Net with 58 patients used for training, the mean values of Dice and TPR in the four parts of the prostate can be considered as the backbone. In terms of overall performance, the effects of the five types of semi-supervised learning have demonstrated growth, among which ICT has the most obvious improvement effect on the overall model. All the semi-supervised learning methods have a higher Dice for region segmentation than supervised learning. However, if we pay attention to the growth percentage of Dice and TPR in the four regions, it can be seen that in EM, CPS, and MT, Dice in a certain region increases, while TPR decreases slightly.

EM is used to reduce the uncertainty of the model and achieves better performance in the TZ region where the morphology is more regular. In the AFS region, where the proportion of pixels is small and the morphology is irregular, making the prediction contour more closely fit the real labels may lead to some changes in the prediction values that exist within the labels themselves, and the small proportion of pixels in AFS can easily lead to the result of elevated Dice and decreased TPR. CPS uses models with the same initialization structure and shows better performance for the PZ and AFS with irregular morphology. The relative pixel ratio of the DPU is very small, while the PZ has the best segmentation effect. The perturbation of the added data in the MT performs better in DPUs with a smaller number of pixels and more regular morphology. The random noise in the TZ region, which introduces some uncertainty during training, may be easily recognized in the TZ as being in the region of the DPU inside it, and a much larger increase in the TPR of the region of the DPU can be seen. However, the edges of the model are closer to the labels, which may also lead to a decrease in the detection of true positives in some regions.

The trade-off for U-Net to obtain more accurate segmentation results for the performance of the model is the need to combine Dice and CE loss when choosing the loss function. So, we can ignore the small amount of TPR in a single region. UAMT is a Monte Carlo uncertainty estimation used in combination with the MT. The addition of this uncertainty estimate improves the accuracy of the prediction value between the TZ and PZ regions and makes the profile of the segmentation results close to the true labels. Notably, this has a high impact on TPR. The strength of ICT lies in the irregular PZ and AFS and becomes the most effective semi-supervised model of the five, with an average increase of 3.55% in Dice and 4.61% in TPR in the overall performance.

The UATS approach achieves the highest overall TPR improvement of 4.77%. It can be seen that the SOTA in prostate semi-supervised learning is still more applicable to the proposed U-Net as a backbone and shows good performance.

On the other hand, in ViT with 58 patients used for training, the effect of the semi-supervised model across all five methods is very small in terms of the overall performance of the model. A decline in performance in certain regions is also observed, even if the overall effect has been improved. Each region is noted to be unstable, but UAMT and CPS demonstrate a great improvement for TPR, while ICT improves in terms of both the overall performance and specific prostate regions. In a framework like ViT that splits images into patches before putting them into the network, simply adding noise can affect the performance of the network. UATS in ViT as the backbone in the AFS is lower in number and the effect of segmentation decreases more for morphological irregularities. This approach is limited with ViT as the backbone.

We added labeled data to our study in an attempt to obtain better image segmentation results in semi-supervised learning. The results show that the segmentation effect of the U-Net backbone network is significantly improved as the amount of labeled data increases. However, the improvement in semi-supervised learning compared to supervised learning decreases, suggesting that when the amount of labeled data is sufficient, semi-supervised learning does not provide a significant improvement in performance. Although for ViT, segmentation is improved by adding training data, the overall performance is still not good. Therefore, simply dividing images into patch embeddings as input has certain requirements on the amount of data in medical images and is not applicable when the amount of labeled data is small.

In the ViT fully supervised case, the tissue region below the prostate is incorrectly identified as a prostate region, and there is no improvement in the semi-supervision learning. Therefore, in the field of prostate segmentation, if semi-supervised learning is added to the training, it is necessary that the fully supervised network is robust for the overall benefit of the model and that the supervised part of learning does not require a very large amount of data. And it also confirms that semi-supervised learning can produce more accurate results for better backbones [16] that remain consistent.

There are some limitations to this comparative study: in the publicly available dataset containing the four regions of the prostate gland segmented, we only found the ProstateX dataset. Therefore, our semi-supervised methods were only experimented on using this dataset. There are many more semi-supervised learning methods available, and we only compared the segmentation results of the more commonly used methods applied to the prostate.

## 5. Conclusions

In this study, after comparing U-Net and ViT, it can be seen that U-Net is more suitable as a backbone in the semi-supervised segmentation of the prostate. Although ViT has achieved great success in natural image data segmentation, it is difficult to demonstrate its performance in medical image data with its morphological complexity and similarity between tissues and small numbers. In semi-supervised learning methods, the UAMT is very effective in improving the accuracy of the segmentation model. With U-Net as a backbone, the TPR of the TZ region increases by 3.99%, the TPR of the DPU region increases by 9.08%, and the overall TPR increases by 9.08%. With ViT as a backbone, the TZ region increases by 2.62%, the AFS region increases by 20.7%, and the overall improvement is 4.53%. Thus, UAMT can be the first choice for improving the accuracy of regional segmentation. ICT typically involves interpolating between the predictions of a model to ensure that similar points in the data distribution have similar predictions. This improves in terms of both the overall and regional performance. The SOTA prostate semi-supervised segmentation method UATS is more applicable to U-Net as the backbone. With U-Net as the backbone, the Dice of the AFS region improved by 12.37% while TPR increased by 12.48%. In terms of overall performance, Dice improved by 3.55% while TPR increased by 4.61%. With ViT as the backbone, Dice and TPR improved in every region. For the stable improvement of the segmentation effect of every region, ICT is preferred. Thus, for regional segmentation, ICT can be the first choice for semi-supervised methods.

## Figures and Tables

**Table 1:** Dice (%) and TPR (%) of the four regions of the prostate using supervised and semi-supervised segmentation methods and their mean values with 58 patients used for training.

Backbone		PZ		TZ		DPU		AFS		Mean	
		Dice	TPR	Dice	TPR	Dice	TPR	Dice	TPR	Dice	TPR
U-Net	Supervised	76.59	73.65	85.99	85.01	72.22	70.43	42.61	49.05	69.35	69.54
	EM	78.09	74.66	87.75	86.40	74.07	73.57	45.33	48.69	71.31	70.83
	CPS	79.68	81.77	87.74	86.04	72.64	70.12	46.42	52.94	71.62	72.72
	MT	77.96	75.39	86.89	84.92	74.35	74.71	43.78	50.73	70.75	71.44
	UAMT	77.71	74.84	86.02	88.40	73.13	77.33	44.09	50.40	70.24	72.74
	ICT	78.87	78.08	87.75	86.37	72.77	71.35	47.88	55.17	71.82	72.74
	UATS	78.90	76.73	87.56	89.61	73.09	73.56	43.60	51.52	70.79	72.86
ViT	Supervised	69.49	70.35	82.68	80.06	61.86	56.75	37.79	38.36	62.96	61.38
	EM	67.61	68.91	81.46	79.89	57.75	49.95	35.88	39.20	60.68	59.49
	CPS	69.34	72.98	82.31	79.30	61.99	60.19	38.30	41.96	62.99	63.61
	MT	69.95	72.91	83.18	79.65	61.97	57.42	33.64	30.99	62.19	60.24
	UAMT	70.54	72.25	82.95	82.16	61.42	55.93	38.39	46.30	63.33	64.16
	ICT	69.59	73.49	82.72	80.89	62.34	58.38	37.28	38.36	62.98	62.78
	UATS	70.43	70.11	83.92	83.21	61.33	58.10	35.88	33.29	62.89	61.18

**Table 2:** Percentage increase in Dice and TPR when semi-supervised learning is used in comparison to supervised learning for the four regions with 58 patients used for training.

Backbone		PZ		TZ		DPU		AFS		Mean	
		Dice (%)	TPR (%)	Dice (%)	TPR (%)	Dice (%)	TPR (%)	Dice (%)	TPR (%)	Dice (%)	TPR (%)
	EM	1.96	1.37	2.05	1.64	2.56	4.46	6.38	−0.73	2.82	1.86
	CPS	4.03	11.03	2.04	1.21	0.58	−0.44	8.94	7.93	3.27	4.58
U-Net	MT	1.79	2.36	1.05	−0.11	2.95	6.08	2.75	3.43	2.01	2.74
	UAMT	1.46	1.62	0.03	3.99	1.26	9.80	3.47	2.75	1.28	4.61
	ICT	2.98	6.01	2.05	1.60	0.76	1.31	12.37	12.48	3.55	4.61
	UATS	3.02	4.18	1.83	5.41	1.2	4.44	2.32	5.04	2.08	4.77
	EM	−2.05	−2.05	−1.48	−0.21	−6.64	−11.98	−5.05	2.19	−3.62	−3.08
	CPS	−0.22	3.74	−0.45	−0.95	0.21	6.06	1.35	9.38	0.05	3.63
ViT	MT	0.66	3.64	0.60	−0.51	0.18	1.18	−10.98	−19.21	−1.22	−1.85
	UAMT	1.51	2.70	0.33	2.62	−0.89	−1.44	1.59	20.70	0.59	4.53
	ICT	0.14	4.46	0.05	1.04	0.78	2.87	1.35	0.00	0.04	2.28
	UATS	1.35	−0.34	1.50	3.93	−0.86	2.38	−5.05	−13.21	−0.11	−0.33

**Table 3:** Dice (%) and TPR (%) and their averages for supervised and semi-supervised segmentation methods for the four regions of the prostate with 68 patients used for training.

Backbone		PZ		TZ		DPU		AFS		Mean	
		Dice	TPR	Dice	TPR	Dice	TPR	Dice	TPR	Dice	TPR
U-Net	Supervised	78.94	80.23	88.75	87.36	72.49	74.78	39.14	40.58	69.83	70.74
	EM	79.31	81.82	89.66	89.13	70.07	70.93	41.75	49.88	70.20	72.94
	CPS	80.43	81.12	89.63	88.85	71.64	71.27	39.49	47.47	70.30	72.18
	MT	80.47	82.35	88.58	85.95	71.07	71.56	40.18	47.16	70.07	71.76
	UAMT	81.12	82.38	89.36	87.13	71.79	75.09	40.29	47.71	70.64	73.08
	ICT	80.72	83.19	88.99	89.05	72.42	74.06	40.87	46.64	70.75	73.23
	UATS	80.39	83.26	88.66	88.57	68.53	67.89	43.15	51.20	70.18	72.73
ViT	Supervised	69.04	74.95	84.25	82.20	58.66	54.73	36.63	38.52	62.15	62.60
	EM	69.26	77.68	85.19	82.40	60.19	54.06	36.7	34.94	62.84	62.27
	CPS	69.14	74.66	84.58	82.50	57.91	53.14	37.79	44.85	62.35	63.79
	MT	69.23	72.21	85.06	82.69	58.95	51.62	36.52	37.08	62.44	60.90
	UAMT	69.02	77.34	84.73	82.20	59.16	56.82	35.42	38.18	62.08	63.63
	ICT	69.11	73.51	84.53	83.30	59.61	55.56	37.88	42.39	62.78	63.70
	UATS	68.70	74.34	85.14	81.82	60.75	56.36	37.92	43.11	63.13	63.91

**Table 4:** Dice (%) and TPR (%) for classical segmentation methods for the four regions of the prostate.

	PZ		TZ		DPU		AFS	
	Dice	TPR	Dice	TPR	Dice	TPR	Dice	TPR
MRF	13.77	25.78	18.92	46.86	0.04	2.23	8.56	34.37
Mean Shift	9.06	19.21	14.15	30.21	0.07	3.31	6.36	22.51
Ostu	9.44	20.15	13.52	45.04	0	0	8.43	22.23

## Data Availability

Data are available at: https://aapm.org/GrandChallenge/PROSTATEx-2/ (accessed on 5 June 2017).
